# A Dual Nano-Signal Probe-Based Electrochemical Immunosensor for the Simultaneous Detection of Two Biomarkers in Gastric Cancer

**DOI:** 10.3390/bios15020080

**Published:** 2025-01-31

**Authors:** Li-Ting Su, Zhen-Qing Yang, Hua-Ping Peng, Ai-Lin Liu

**Affiliations:** 1 Quanzhou Medical College, Quanzhou 362000, China; 2005009@qzmc.edu.cn; 2 The School of Pharmacy, Fujian Medical University, Fuzhou 350122, China; lbe_yzq@accube.cn

**Keywords:** dual biomarkers, dual nano-signal probe, electrochemical immunosensor

## Abstract

Detecting multiple tumor markers is of great importance. It helps in early cancer detection, accurate diagnosis, and monitoring treatment. In this work, gold nanoparticles–toluidine blue–graphene oxide (AuNPs-TB–GO) and gold nanoparticles–carboxyl ferrocene–tungsten disulfide (AuNPs–FMC–WS_2_) nanocomposites were prepared for labeling Carcinoembryonic antigen (CEA) antibody and Carbohydrate antigen 72–4 (CA72-4) antibody, respectively, and used as two kinds of probes with different electrochemical signals. With the excellent magnetic performance of biotin immune magnetic beads (IMBs), the biofunctional IMBs were firmly deposited on the magnetic glassy carbon electrode (MGCE) surface by applying a constant magnetic field, and then the CEA and CA72-4 antibody were immobilized on the IMBs by the avidin–biotin conjugation. The assay was based on the change in the detection peak current. Under the optimum experimental conditions, the linear range of detection of CEA is of the two-component immunosensor is from 0.01 to 120 ng/mL, with a low detection limit of 0.003 ng/mL, and the linear range of detection of CA72-4 is from 0.05 to 35 U/mL, with a detection limit of 0.016 U/mL. The results showed that the proposed immunosensor enabled simultaneous monitoring of CEA and CA72-4 and exhibited good reproducibility, excellent high selectivity, and sensitivity. In particular, the proposed multiplexed immunoassay approach does not require sophisticated fabrication and is well-suited for high-throughput biosensing and application to other areas.

## 1. Introduction

Gastric cancer (GC) is one of the most common cancers and the third leading cause of cancer-related death globally, making early diagnosis crucial for improving the overall treatment outcomes for gastric cancer patients [1,2,3]. However, the diagnosis and treatment of gastric cancer still face significant challenges due to its numerous heterogeneities and complex pathogenesis [4]. Currently, the early diagnosis of gastric cancer mainly relies on upper gastrointestinal endoscopy combined with tissue biopsy, serum gastric function analysis, ultrasound, and radiological examinations [5]. Nevertheless, these means may be limited by high costs, time-consuming procedures, complex equipment, and invasive biopsies [6]. The screening and detection of tumor markers are some of the most effective methods for early cancer detection and prognosis [7]. The commonly used clinical methods for tumor marker detection mainly include electrochemiluminescence, in addition to radioimmunoassay, fluorescence immunoassay, Polymerase Chain Reaction (PCR) and its derivative technologies, as well as biosensor technology [8,9,10]. Electrochemical immunosensors that combine immunoassay techniques with electrochemical sensors are promising tools for tumor marker analysis due to their advantages of high sensitivity, good selectivity, cost-effectiveness, and rapid results [11,12,13,14].

At present, the commonly used serum tumor markers for gastric cancer diagnosis include CEA, CA72-4, carbohydrate antigen 19-9 (CA19-9), and pepsinogen (PG), etc. [15]. Although tumor markers are highly correlated with tumor typing, a single tumor marker cannot specifically indicate tumor types [16,17]. The multi-component joint detection immunoassay technology, which detects multiple tumor markers, can significantly improve the accuracy and sensitivity of cancer diagnosis and is of great significance for clinical diagnosis, disease assessment, and effective monitoring of treatment [18,19,20]. In the meantime, the simultaneous determination of multiple tumor markers is more advantageous than single-component detection, including high analytical throughput, shorter needed time, and reduced sample consumption, thus reducing the analytical costs [21]. Therefore, to meet clinical testing demands, developing high-throughput analytical methods that can process and analyze multiple samples in a single procedure is a long-term goal and research focus in tumor diagnosis [22,23].

**Figure 1:**
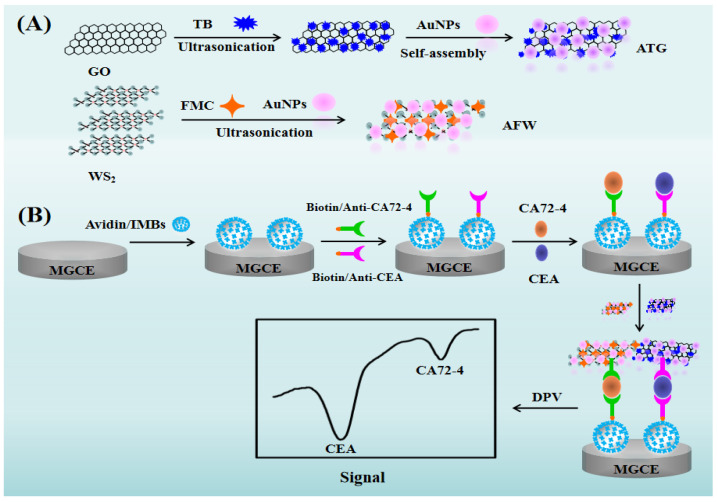
(**A**) Schematic representation of the synthesis of the ATG nanocomposite and AFW nanocomposite. (**B**) Schematic illustration of the electrochemical immunosensor for the simultaneous detection of CEA and CA72-4.

Nanomaterials, such as carbon-based nanomaterials (carbon nanotubes, graphene, carbon spheres, etc.) [24,25], metal nanomaterials (gold nanoparticles, platinum nanoparticles, silver nanomaterials, etc.) [26,27], and metal oxide nanomaterials (silica, iron oxide, zinc oxide, etc.) [28,29], have become some of the popular materials for research and application due to their unique properties in terms of optical, magnetic, electrical, and catalytic performance. Among them, GO has advantages such as a large specific surface area and strong electrical conductivity [30]. Its structure contains functional groups, enabling it to be easily modified. Thus, many GO-based nanomaterials can be synthesized and have been widely and effectively applied in various fields, including batteries, catalysis, sensors, and cell and drug analysis [31]. AuNPs have stable properties, excellent optoelectronic performance, and biocompatibility [32], as well as a high specific surface area. The surface of AuNPs is negatively charged, allowing them to bind to positively charged substances through electrostatic forces or to form nanocomposites with excellent properties with numerous compounds by utilizing Au-N or Au-S bonds [33]. As a layered transition-metal chalcogenide, WS_2_ has a special two-dimensional ultra-thin atomic layer structure, unique physical, optical, and electrical properties, and excellent electrochemical performance, especially in the research and application of electrode materials, catalysts, supercapacitors, and sensors [34]. Both TB and FMC have good redox activity, electrical conductivity, and high stability [35,36]. As shown in Figure 1, we synthesized AuNPs-TB-GO (abbreviated as ATG) and AuNPs-FMC-WS_2_ (abbreviated as AFW) through ultrasonic and oscillation methods. These two nanocomposites combine the advantages of various materials such as GO, WS_2_, TB, FMC, and AuNPs, which is conducive to the subsequent construction of immunosensors and the effective detection of gastric cancer dual biomarkers.

During this construction of immunosensors, antibody labeling technology is one of the key technologies in multi-component immunoassay, where the distinguishability of the signals and the labeling method determine the effectiveness of multi-component analysis [37]. We have selected two electroactive substances, TB and FMC, as antibody markers and used the significantly different redox peak potentials of them to distinguish the two antibodies. As shown in Figure 1, ATG and AFW were labeled with Anti-CEA secondary antibody (CEA-Ab_2_) and Anti-CA72-4 secondary antibody (CA72-4-Ab_2_), respectively, and used them as different nano signal probes, namely gold nanoparticles–toluidine blue–graphene oxide-Carcinoembryonic antigen-secondary antibody (ATG-CEA-Ab_2_) and gold nanoparticles–carboxyl ferrocene–tungsten disulfide-Carbohydrate antigen 72-4 secondary antibody (AFW-CA72-4-Ab_2_). Meanwhile, using IMBs with excellent magnetic properties as the primary antibody immobilization substrate, the magnetic beads were firmly fixed on the electrode surface through the action of an external magnetic field. Then, by utilizing the affinity bond between biotin and avidin, the effective immobilization of biotinylated Anti-CEA primary antibody (CEA-Ab_1_) and biotinylated Anti-CA72-4 primary antibody (CA72-4-Ab_1_) on the electrode surface was achieved, thereby successfully constructing a simple and efficient two-component immunosensing interface. On the basis of the above sensing interface, through further sandwich immunoassay, the secondary antibody composite nanomaterials labeled with different signals were combined on the surface of the immunosensor, thus achieving highly sensitive simultaneous detection of gastric cancer markers CEA and CA72-4. Given its high sensitivity, the developed biosensor has excellent specificity and rapid responsiveness and is expected to contribute to the joint detection of gastric cancer biomarkers.

## 2. Materials and Methods

### 2.1. Chemicals and Reagents

GO was purchased from Nanjing Xianfeng Nanomaterial Technology Company. TB, FMC, and WS_2_ (2 μm, 99% purity) and bovine serum albumin (BSA) were purchased from Sigma-Aldrich (St. Louis, MO, USA). Chloroauric acid, trisodium citrate, and tannic acid were purchased from Shanghai National Pharmaceutical Group Chemical Reagent Company. IMBs, CEA antigen (CEA-Ag), CEA-Ab_1_, CEA-Ab_2_, CA72-4 antigen (CA72-4-Ag), CA72-4-Ab_1_, and CA72-4-Ab_2_ were purchased from Roche Diagnostics GmbH, Germany. Phosphate buffer solutions containing 0.1 M KCl were used as the electrolyte (PBS, 0.1 M). All reagents were of analytical grade, and double-distilled water was used throughout the experiments.

### 2.2. Instruments and Apparatus

Ultraviolet–visible (UV–vis) absorption spectrum were performed on a UV-2450 spectrophotometer (Shimadzu Corporation, Kyoto, Japan). Transmission electron microscopy (TEM) and Energy dispersive X-ray (EDX) spectrometry were characterized by JEOL 2010 (JEOL, Tokyo, Japan). Autolab PGSTAT302F electrochemical workstation (Metrohm, Netherlands), JEM 2100 Transmission Electron Microscope (Hitachi, Tokyo, Japan). Electrochemical experiments for cyclic voltammetry (CV) and differential pulse voltammetry (DPV) were performed on a CHI630C electrochemical workstation (Shanghai Chenhua Instruments Co., Shanghai, China) using a conventional three-electrode system, which consisted of a modified magnetic glassy carbon electrode (MGCE, Ø3mm) (Tianjin Gaoshirui Lian Technology Company, Tianjin, China) as the working electrode, a platinum wire electrode as the counter electrode, and Ag/AgCl as the reference electrode. The electrochemical impedance spectroscopy (EIS) measurement was performed on an Autolab PGSTAT30 electrochemical workstation (Metrohm, The Netherlands).

### 2.3. Synthesis of AuNPs–TB–GO

Gold nanoparticles (AuNPs) were prepared via a previously reported sodium citrate reduction method [38]. Briefly, 1 mL of 2.0% chloroauric acid solution and 79 mL of distilled water were added to a flask A. Subsequently, 8 mL of 1.0% trisodium citrate and 0.2 mL of 1.0% tannic acid were added to flask B, followed by the addition of 11.8 mL of double-distilled water. Both flasks were heated to 60 °C, and the solution in flask B was rapidly poured into flask A. The mixture was vigorously stirred at 60 °C for 35 min until the color turned purple-red. After cooling to room temperature, the synthesized AuNPs were stored in the dark at 4 °C.

Additionally, a certain amount of GO solid was dispersed in double-distilled water, and after 1 h of ultrasonication, the mixture was centrifuged at 10,000 rpm for 10 min. The supernatant was collected to prepare a 2 mg/mL brownish-yellow GO solution. Then, 10 mg/mL TB solution was added dropwise to 500 μL of the GO solution under sonication, and the solution color changed from brown to green, finally to blue. The blue mixture was centrifuged at 10,000 rpm for 10 min and washed three times with double-distilled water; the obtained precipitate was dissolved in 1 mL of water named TB-GO composite.

Subsequently, 200 μL of AuNPs solution was added to 100 μL of TB-GO composite; the mixture was shaken for 1 h and then soaked overnight. Then, the mixture was washed using a 1:1 volume ratio of water and ethanol solution, followed by centrifugation at 10,000 rpm for 10 min. The precipitate was collected and dispersed in 1 mL of water, and this AuNPs-TB-GO composite was named as ATG.

### 2.4. The Synthesis of ATG–CEA–Ab_2_

1 mL of CEA secondary antibody was added into 1 mL of ATG composite and reacted under agitation at 180 rpm at 37 °C for 20 min. After washing several times, 2 mL of 0.5% BSA PBS solution was added to the mixture, and it was stirred at room temperature for 1 h to block the surface of unbound ATG with CEA. The supernatant was discarded, and this washing process was repeated three times. Finally, 1 mL of PBS solution was added to the product, and the dispersion was stored at 4 °C.

### 2.5. Synthesis of AuNPs-FMC-WS_2_ Composite

AFW composites were prepared using a one-step ultrasonic chemical method [39]. A total of 100 mg WS_2_ and 50 mg FMC were weighed, then water was added to 20 mL, and they were mixed and sonicated. Then, 2 mL AuNPs solution was added to this mixture, and sonication continued for 6 h. Subsequently, the resulting mixture was centrifuged at 10,000 rpm for 10 min, the supernatant was collected, and then it was centrifuged again at 16,000 rpm for 10 min. The obtained precipitate was dispersed in 1 mL double-distilled water and washed with a mixture of water and ethanol (1:1). After centrifugation, the precipitate was collected, and it was dispersed in 1 mL double-distilled water, and it was named AFW. Likewise, under the same condition without adding gold nanoparticles, the FMC-WS_2_ composite can be prepared.

### 2.6. Preparation of AFW-CA72-4-Ab_2_

1 mL AFW was mixed with 1 mL 6 μg/mL CA72-4-Ab2 and allowed to react under agitation at 180 rpm at 37 °C for 20 min. After having been washed several times, 2 mL of 0.5% BSA PBS solution was added and stirred at room temperature for 1 h to block the surface of unbound AFW with CA72-4 [39]. The supernatant was discarded. This wash process was repeated three times. Finally, 1 mL PBS solution was added to the product, and this dispersion was stored at 4 °C.

### 2.7. Construction of Dual-Component Immunosensor

Firstly, the MGCE was polished sequentially on chamois with 1.0 μm, 0.3 μm, and 0.05 μm Al_2_O_3_. Then, the polished MGCE was cleaned under sonication (approximately 3 min for each) using the mixture of HNO_3_ and water (1:1), anhydrous ethanol, and distilled water, respectively. After rinsing with double-distilled water, it was dried with high-purity N_2_. A total of 6 μL of IMBs solution was dropped onto the MGCE surface, and it was dried in a vacuum for 30 min. Subsequently, equal volumes of CEA-Ab_1_ and CA72-4-Ab_1_ were dropped onto the electrode surface and left overnight (4 °C). The electrode was then washed with PBS at pH 7.4 and dried with N_2_. Finally, equal volumes of CEA antigen and CA72-4 antigen mixture were dropped onto the electrode surface, incubated at 37 °C for 1 h, and washed with PBS.

Additionally, a mixture solution of ATG-CEA-Ab_2_ and AFW-CA72-4-Ab_2_ was dropped onto the electrode surface and incubated at 37 °C for 1 h, followed by washing with PBS. The modified MGCE served as the working electrode, a platinum wire electrode as the counter electrode, and Ag/AgCl as the reference electrode. Before the measurement, N_2_ was purged for at least 20 min to remove O_2_ from the solution. Then, the DPV scans were performed in PBS buffer solution at pH 7.4, with a scan potential range of −0.6 to +0.6 V and a scan rate of 100 mV/s. The quantitative analysis of the target antigen was conducted based on the change in oxidation peak current values.

## 3. Results and Discussions

### 3.1. The Characterization of Nano Materials

**Figure 2:**
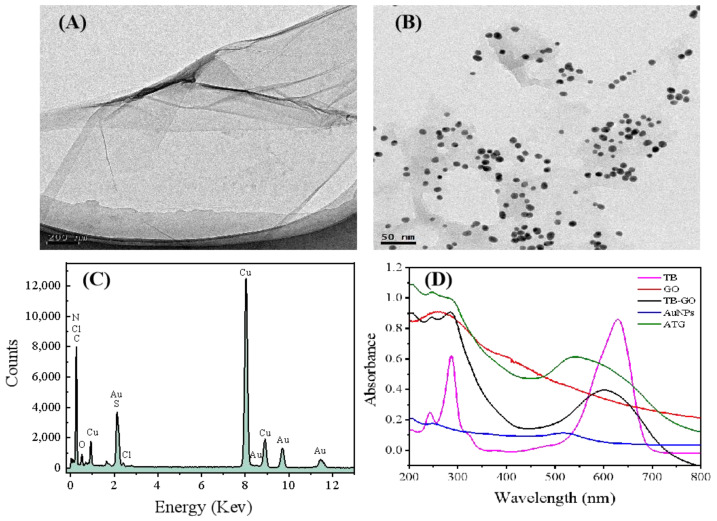
TEM images of (**A**) TB-GO, (**B**) AuNPs-TB-GO. (**C**) EDX spectrum of AuNPs-TB-GO composite. (**D**) UV-vis spectra of TB (pink line), GO (red line), TB-GO (black line), Au NPs (blue line), ATG (green line).

The morphology and preparation process of ATG composite were confirmed by TEM. As shown in Figure 2A, TB-GO exhibits a sheet-like structure, indicating that GO has been successfully exfoliated through ultrasonic treatment. It is obvious to see that a large number of spherical nanoparticles are uniformly loaded on the surface of the TB-GO composite, suggesting that AuNPs have been self-assembled onto the TB-GO composite surface through electrostatic interactions, forming the ATG composite (Figure 2B, Figure S1A,B). Moreover, EDX characterization reveals that the composite contains elements such as C, Au, N, and S, further confirming the successful self-assembly of the nano ATG composite (Figure 2C). In addition, UV-vis spectroscopy was employed for further characterization of the ATG nanocomposite. As depicted in Figure 2D, TB shows two absorption peaks at 288 nm and 632 nm. GO has a characteristic absorption peak at 228 nm; TB-GO nanocomposite presents both characteristic absorption peaks of GO and TB. Compared to TB, TB-GO undergoes a blue shift due to steric hindrance during the formation of conjugated structures between TB and GO, resulting in a characteristic absorption peak at 598 nm, indicating the successful immobilization of TB onto graphene. Moreover, AuNPs display an absorption peak at 520 nm; upon combination with TB-GO, the prepared AuNPs-TB-GO nanocomposite exhibits a broad absorption peak around 540 nm due to interactions between AuNPs and TB. These results further confirm the successful preparation of the ATG nanocomposite.

**Figure 3:**
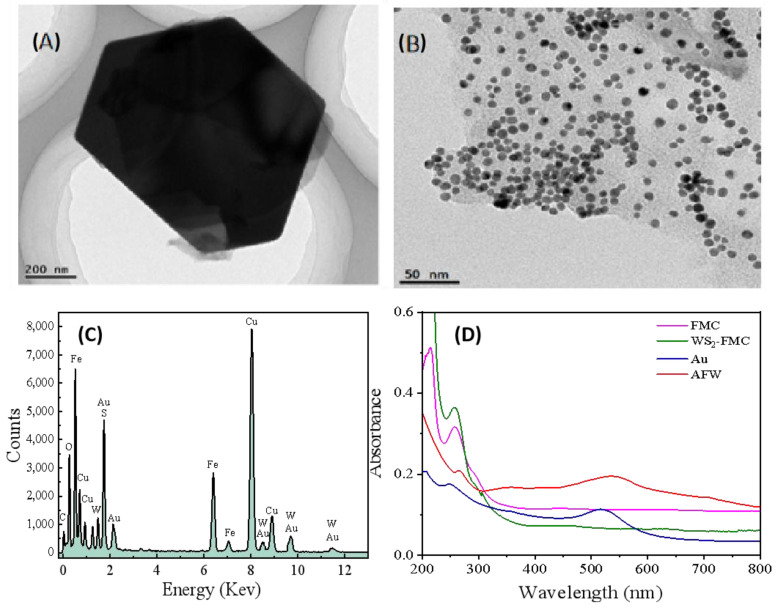
TEM images of (**A**) bulk WS_2_, (**B**) AFW nanocomposite. (**C**) EDX spectrum of AFW. (**D**) UV-vis spectra of FMC (pink line), WS_2_-FMC (green line), Au (blue line), AFW (red line).

TEM analysis was also utilized to verify the morphology and synthesis process of AFW. As shown in Figure 3A, the purchased WS_2_ exhibits a multilayer structure; however, after mixing with FMC and AuNPs followed by ultrasonication, there were numerous AuNPs on the sheet-like surface of WS_2_ (Figure 3B). It can be observed that during the ultrasonication process, WS_2_ has been exfoliated from a multilayer structure into a sheet-like structure. This could also be seen from Figure S2. Additionally, EDX characterization indicates that W, S, Au, Fe, and C elements appeared in this composite, confirming that FMC and AuNPs have been successfully self-assembled on WS_2_ nanosheets (Figure 3C). Therefore, these results demonstrate that AuNPs-FMC-WS_2_ composites can be successfully synthesized via a simple ultrasonication procedure. We also use UV-vis spectroscopy to further characterize the AFW nanocomposite. As shown in Figure 3D, pure FMC exhibits a characteristic absorption peak at 256 nm, while the WS_2_-FMC composite, in addition to the peak at 256 nm, shows new absorption peaks at 450 nm and 625 nm, respectively. This indicates successful loading of FMC onto the surface of WS_2_ through coordination interactions. Furthermore, a new absorption peak at 540 nm appeared in Figure 3D, which undergoes a redshift compared to the absorption peak of pure AuNPs at 520 nm due to the interactions between AuNPs and WS_2_, suggesting that AFW nanocomposite has been successfully prepared.

### 3.2. The Electrochemical Characterization of Modified Electrodes

**Figure 4:**
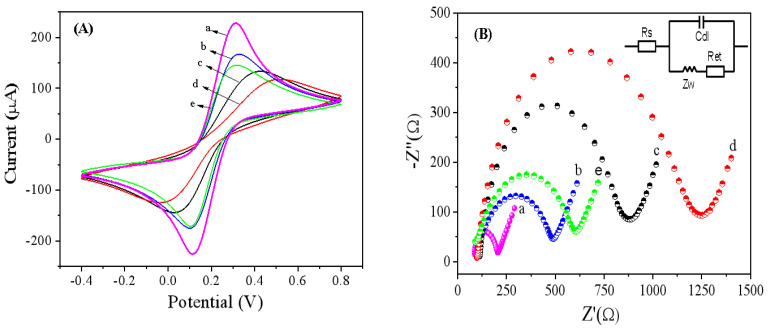
(**A**) The cyclic voltammograms of different modified magnetic carbon glassy electrodes with the voltage range from −0.4 V to 0.8 V; the scan rate was 100 mV/s in a 0.1 M KCl solution containing 10 mM K_3_Fe(CN)_6_. (**B**) The electrochemical impedance spectroscopy of different modified magnetic carbon glassy electrodes was performed in a 0.1 M KCl solution containing 10 mM Fe(CN)6^4−^/^3−^ at an open potential of 210 mV within the frequency range of 0.01 Hz to 100 kHz. (a) bare MGCE, (b) IMBs/MGCE, (c) CEA-Ab_1_&CA72-4-Ab_1_/IMBs/MGCE, (d) CEA-Ag&CA72-4-Ag/CEA-Ab_1_&CA72-4-Ab_1_/IMBs/MGCE, (e) ATG-CEA-Ab_2_&AFW-CA72-4- Ab_2_/CEA-Ag&CA72-4-Ag/CEA-Ab_1_&CA72-4-Ab_1_/IMBs/MGCE.

In this article, cyclic voltammetry (CV) and electrochemical impedance spectroscopy (EIS) were employed to characterize the electrochemical behavior of the modified electrodes during the sensor modification process. Firstly, cyclic voltammetry was used to characterize the assembly process of the immunosensor. The electrochemical properties of different modified electrodes were investigated. Figure 4A shows the cyclic voltammetry curves of different modified electrodes with the voltage range from −0.4 V to 0.8 V; the scan rate was 100 mV/s in a 0.1 M KCl solution containing 10 mM K_3_Fe(CN)_6_. A pair of obvious oxidation–reduction peaks was found when K_3_Fe(CN)_6_ was on the bare MGCE (curve a). Clearly, the immobilized IMBs on the electrode surface could hinder the electron diffusion to the electrode, resulting in a decrease in the oxidation–reduction peaks of IMBs/MGCE (curve b) compared to those of bare MGCE. When the mixture of CEA-Ab_1_ and CA72-4-Ab_1_ is immobilized onto the MGCE surface by the affinity between biotin and avidin, the oxidation–reduction peak current is smaller than that of IMBs/MGCE (curve c), which is consistent with the fact that proteins hinder electron transfer on the electrode surface. Similarly, after further incubation of the mixture of CEA and CA72-4 antigens on the electrode, the resulting curve (curve d) shows a further decrease in the oxidation–reduction current. However, when the mixed solution of ATG-CEA-Ab_2_ and AFW-CA72-4-Ab_2_ is specifically bound to the CEA and CA72-4 antigens on the electrode surface, the oxidation–reduction current increases (curve e). The reasons may be as follows: (1) The good conductivity of nanoprobes could accelerate the electron transfer on the electrode surface; (2) the positively charged electron mediator TB in the AuNPs-TB-GO composite is able to effectively adsorb negative [Fe(CN)_6_]^3−^ to the electrode surface, leading to a significant increase in [Fe(CN)_6_]^3−^ peak current.

In Figure 4B, the changes in the electron transfer resistance (Ret) on different modified electrode surfaces were detected by electrochemical impedance spectroscopy when the bare MGCE, IMBs/MGCE, CEA-Ab_1_&CA72-4-Ab_1_//IMBs/MGCE, CEA-Ag&CA72-4Ag/Ab_1_/IMBs/MGCE, and ATG-CEA-Ab_2_&AFW-CA72-4-Ab_2_/CEA-Ag &CA72-4-Ag/CEA-Ab_1_&CA72-4-Ab_1_/IMBs/MGCE were placed in a 0.1 M KCl solution containing 10 mM Fe(CN)_6_^4−/3−^. The result indicated that due to the semiconducting property of the immunomagnetic beads (IMBs) and the biotin on the magnetic bead surface, it could hinder electron transfer, resulting in a larger Ret of IMBs/MGCE (curve b) than that of the bare MGCE (curve a). When the biotinylated CEA-Ab_1_ and CA72-4-Ab_1_ mixture is immobilized onto the MGCE surface by affinity, the resistance significantly increases (curve c), due to the fact that antibodies are large biomolecules that also hinder electron transfer. Similarly, when the mixture of CEA and CA72-4 antigens is further incubated on the electrode, the resulting immunocomplex further hinders electron transfer, leading to a further increase in Ret (curve d). Nevertheless, when the nanoprobes ATG-CEA-Ab_2_ and AFW-CA72-4-Ab_2_ are specifically bound to the CEA and CA72-4 antigens immobilized on the electrode surface, Ret decreases after immunoreaction (curve e), which is consistent with the results of cyclic voltammetry. Based on the CV and EIS results, it is proved that the assembly of IMBs, Ab_1_, Ag, and Ab_2_ on the electrode surface has been achieved through affinity interactions and specific immune reactions; meanwhile, the biological activity of the CEA&CA72-4 primary antibody immobilized on IMBs/MGCE was effectively maintained. As a result, a dual-component CEA and CA72-4 detection immunosensor has been successfully constructed.

### 3.3. Optimization of Experimental Conditions

**Figure 5:**
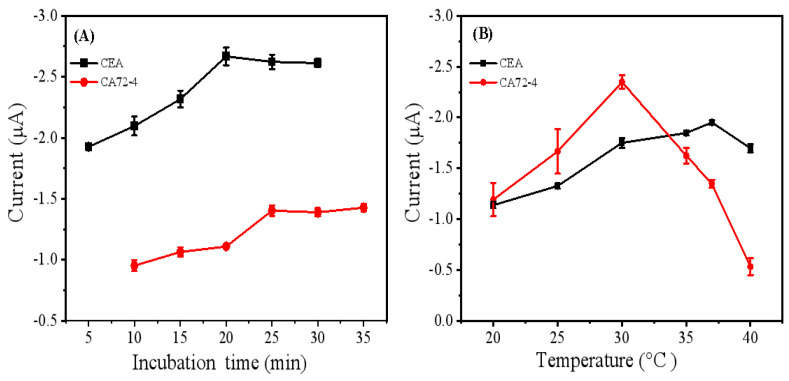
Effect of (**A**) incubation time and (**B**) incubation temperature on the DPV response in the presence of CEA and CA72-4. The DPV scans were performed in PBS buffer solution at pH 7.4, with a scan potential range of −0.6 to +0.6V, a scan rate of 100 mV/s.

To evaluate the performance of this immune sensor, two optimal conditions of incubation time and temperature were confirmed. As shown in Figure 5A, the dual-component DPV response current values increased firstly with a longer incubation time, and after interacting for 20 min, the response current signal remains relatively stable, indicating completion of the immune reaction. In addition, after 25 min of incubation of CA72-4 antigen, an essentially unchanged current value was also observed, suggesting reaching an equilibrium state. Therefore, the optimal incubation time selected for this experiment is 25 min.

In Figure 5B, as the temperature varies from 20 to 40 °C, the current response signal value also changes. It can be observed that the maximum current value for CEA antigen is at 37 °C, while a higher temperature will result in a decrease in current response, since antigen and antibody may gradually inactivate under high temperature. Similarly, the CA72-4 antigen shows the largest current signal value at 30 °C, then decreases with the increase in temperature. Hence, the optimal temperature for the immune reaction is chosen as 35 °C.

### 3.4. Quantitative Analytical Performance

**Figure 6:**
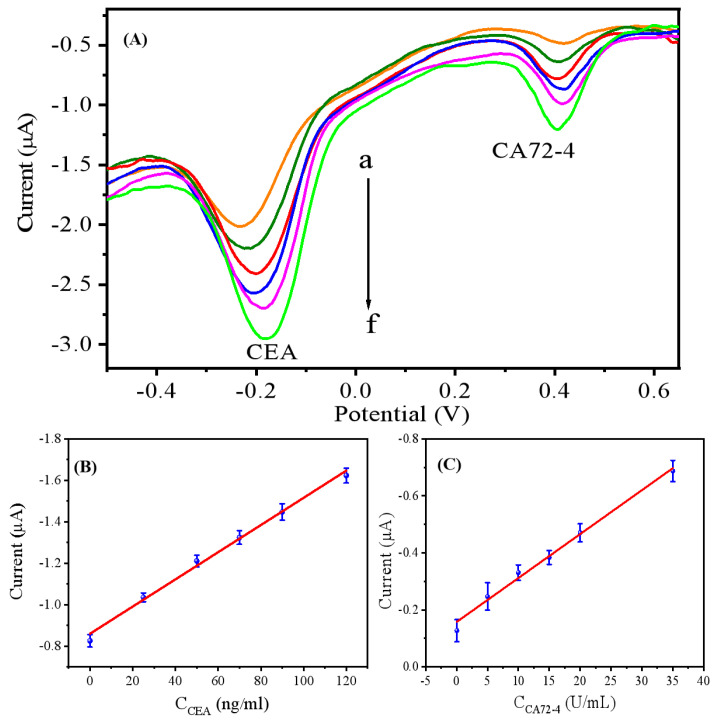
(**A**) DPV responses with different antigen concentrations. Curves a-f corresponding to CEA concentration in the range of 0.01–120 ng/mL and CA72-4 concentration in the range of 0.05–35 U/L. The DPV scans were performed in PBS buffer solution at pH 7.4, with a scan potential range of −0.6 to +0.6 V, a scan rate of 100 mV/s. (**B**) The linear equation of CEA is *I* = −0.006 *C_CEA_*−0.845 (R^2^ = 0.9913). **(C)** The linear equation of CA72-4 is I = −0.0154C_CA72-4_−0.1575 (R^2^ = 0.9871).

Under optimal conditions, the amperometric response of the dual-component immune sensor to both CEA and CA72-4 components was studied by DPV. As shown in Figure 6A, with the increase in antigen concentration, more CEA and CA72-4 molecules specifically bind to Ab_1_ in the sensor. This will lead to more immune complexes on the electrode surface formed by sandwich immunoassays with ATG and AFW labeled CEA and CA72-4 secondary antibodies, respectively, contributing to a continuous increase in the DPV current signal. The results indicate that this dual-component immune sensor exhibits good linear relationships to CEA and CA72-4, which are 0.01~120 ng/mL and 0.05~35 U/mL, respectively. The linear equations are *I* = −0.006 *C_CEA_*−0.845 and *I* = −0.0154*C_CA72-4_*−0.1575, with the linear correlation coefficients of 0.9913 and 0.9871, respectively (Figure 6B,C). The detection limits of the immune sensor for CEA and CA72-4 are 0.003 ng/mL and 0.016 U/mL, with S/N = 3. As shown in Table S1, the detection limits of the biosensor for CEA and CA72-4 are comparable to or better than those of some of the previously reported sensors [40,41,42,43,44,45].

### 3.5. Specificity, Stability, and Reproducibility

**Figure 7:**
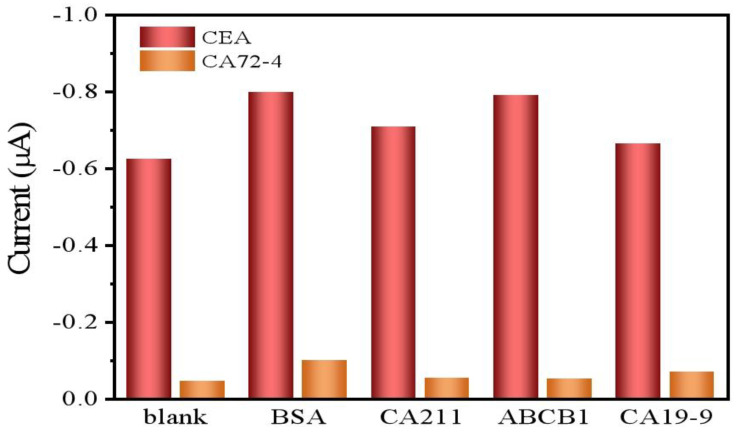
Selectivity of the immune sensor toward BSA, CA211, ABCB1, and CA19-9. The DPV scans were performed in PBS buffer solution at pH 7.4, with a scan potential range of −0.6 to +0.6V, a scan rate of 100 mV/s.

To evaluate the selectivity of this immune sensor, the current values were recorded when the dual-component immune sensor was placed in 10 ng/mL BSA, 10 ng/mL CA211, 10 ng/mL ABCB1, and 10 U/mL CA19-9 solutions, respectively, and incubated for 25 min. In Figure 7,the result showed that there was no significant current value difference compared to the control group (without this interference), demonstrating that the dual-component immune sensor exhibits good anti-interference capability and satisfactory selectivity.

The prepared immune electrode was immersed in a mixture of 25 ng/mL CEA and 10 U/mL CA72-4, and its electrochemical response signal was detected six times. The standard deviations of six measurements of CEA and CA724 were 3.3% and 3.9%, respectively. Additionally, six immune electrodes were used to detect the same mixed sample, resulting in standard deviations of CEA and CA724 of 3.6% and 6.6%, respectively. Furthermore, after storing the prepared immune sensor at 4 °C for 30 days, the magnitudes of the electrical signals of CEA and CA72-4 measured could still maintain 91% and 89% of the initial current, indicating excellent long-term stability of this immune sensor.

### 3.6. Recovery Experiment

To investigate the feasibility of this immune sensor for detecting CEA and CA72-4 in actual samples, spike-recovery experiments were conducted. The serum samples were diluted to 10 times firstly; then diluting it into a series of test solutions using 0.1 mol/L PBS (pH 7.4) solution. In Table 1, the recovery rates of CEA and CA72-4 are between 92.63–102.00% and 94.80–99.44%, respectively, indicating that the immune sensor exhibits high applicability and reliability for detecting CEA and CA72-4 in actual samples.

## 4. Conclusions

In conclusion, we designed and constructed a novel sandwich-type electrochemical immunosensor with dual nano signal probes for the simultaneous detection of CEA and CA72-4 in gastric cancer. On the one hand, this design is novel because avidin-immobilized immunomagnetic beads were utilized as the antibody-carrying substrate and could be facilely immobilized on the MGCE surface only through an external magnetic field. On the other hand, the synthesis method of dual-probe nanocomposites is simple, and by utilizing their unique properties, a sensitive and accurate two-component immunosensing interface has been constructed, achieving low-level detection of CEA and CA724 at 0.003 ng/mL and 0.016 U/mL. This sensor has high sensitivity, good selectivity, reproducibility, and stability. Therefore, it has potential application value in the clinical diagnosis of cancer, providing new ideas for constructing multi-component tumor biomarker sensors.

## Figures and Tables

**Table 1:** Recovery experiment results for detection of CEA and CA72-4 in human serum samples (*n* = 3).

Sample	Added	Mean Value	RSD (%)	Recovery (%)
S1	CEA 1 ng/mL	1.02 ng/mL	2.09	102.00
	CA72-4 5 U/mL	4.84 U/mL	2.42	94.80
S2	CEA 100 ng/mL	99.70 ng/mL	1.74	99.70
	CA72-4 25 U/mL	24.86 U/mL	2.36	99.44
S3	CEA 200 ng/mL	185.26 ng/mL	2.82	92.63
	CA72-4 35 U/mL	34.32 U/mL	1.67	98.06

## Data Availability

The data used in this study are available from the corresponding authors upon reasonable request.

## Supplementary Materials

The following supporting information can be downloaded at: https://www.mdpi.com/article/10.3390/bios15020080/s1, Figure S1: TEM images of (A), (B), AuNPs-TB-GO; Figure S2: TEM images of bulk WS2 after sonication; Table S1: Comparison of LOD about different CEA/CA72-4 detection methods.
