# Supplementary material for: A Dual Nano-Signal Probe-Based Electrochemical Immunosensor for the Simultaneous Detection of Two Biomarkers in Gastric Cancer
Source: Biosensors (Basel). 2025 Jan 31;15(2):80. doi: 10.3390/bios15020080 (PMC11853661; doi:10.3390/bios15020080)
Supplement: Supplementary file 1 [file biosensors-15-00080-s001.zip › biosensors-3323538-supplementary.pdf]

Supporting Information

A Dual Nano-signal Probe-Based Electrochemical Immunosensor for the Simultaneous Detection of Two Biomarkers in Gastric Cancer

Li-Ting Su [1,2], Zhen-Qing Yang [2], Hua-Ping Peng [2,*] and Ai-Lin Liu [2,*]

[1] Quanzhou Medical College, Quanzhou 362000, China; 2005009@qzmc.edu.cn
[2] The School of Pharmacy, Fujian Medical University, Fuzhou 350122, China; 2005009@qzmc.edu.cn
* Correspondence: penghuaping@fjmu.edu.cn (H.-P.P.); ailinliu@fjmu.edu.cn (A.-L.L.)

3.Results and Discussions

3.1 The characterization of nano materials

The morphology and preparation process of ATG composite were confirmed by TEM. As shown in Figure 2A, TB-GO exhibits a sheet-like structure, indicating that GO has been successfully exfoliated through ultrasonic treatment. It is obvious to see that a large number of spherical nanoparticles are uniformly loaded on the surface of the TB-GO composite, suggesting that AuNPs have been self-assembled onto the TB-GO composite surface through electrostatic interactions, forming the ATG composite (Figure 2B, **Figures S1A,B**). Moreover, EDX characterization reveals that the composite contains elements such as C, Au, N, and S, further confirming the successful self-assembly of the nano ATG composite (Figure 2C). In addition, UV-vis spectroscopy was employed for further characterization of the ATG nanocomposite. As depicted in Figure 2D, TB shows two absorption peaks at 288 nm and 632 nm. GO has a characteristic absorption peak at 228 nm; TB-GO nanocomposite presents both characteristic absorption peaks of GO and TB. Compared to TB, TB-GO undergoes a blue shift due to steric hindrance during the formation of conjugated structures between TB and GO, resulting in a characteristic absorption peak at 598 nm, indicating the successful immobilization of TB onto graphene. Moreover, AuNPs display an absorption peak at 520 nm; upon combination with TB-GO, the prepared AuNPs-TB-GO nanocomposite exhibits a broad absorption peak around 540 nm due to interactions between AuNPs and TB. These results further confirm the successful preparation of the ATG nanocomposite

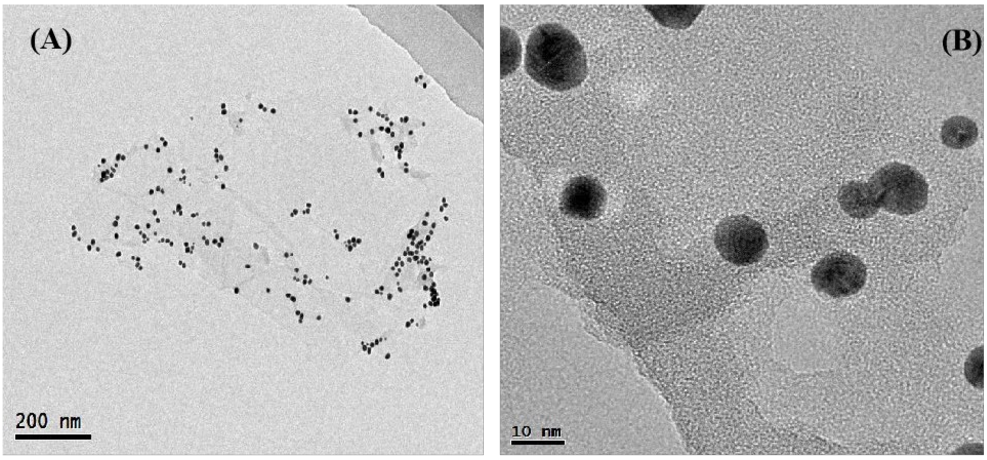

Figure S1. TEM images of (A), (B), AuNPs-TB-GO.

TEM analysis was also utilized to verify the morphology and synthesis process of AFW. As shown in Figure 3A, the purchased WS_2 exhibits a multilayer structure; however, after mixing with FMC and AuNPs followed by ultrasonication, there were nu-

merous AuNPs on the sheet-like surface of WS_2 (Figure 3B). It can be observed that during the ultrasonication process, WS_2 has been exfoliated from a multilayer structure into a sheet-like structure. This could also be seen from **Figure S2**. Additionally, EDX characterization indicates that W, S, Au, Fe, and C elements appeared in this composite, confirming that FMC and AuNPs have been successfully self-assembled on WS_2 nanosheets (Figure 3C). Therefore, these results demonstrate that AuNPs-FMC-WS_2 composites can be successfully synthesized via a simple ultrasonication procedure. We also use UV-vis spectroscopy to further characterize the AFW nanocomposite. As shown in Figure 3D, pure FMC exhibits a characteristic absorption peak at 256 nm, while the WS_2-FMC composite, in addition to the peak at 256 nm, shows new absorption peaks at 450 nm and 625 nm, respectively. This indicates successful loading of FMC onto the surface of WS_2 through coordination interactions. Furthermore, a new absorption peak at 540 nm appeared in Figure 3D, which undergoes a redshift compared to the absorption peak of pure AuNPs at 520 nm due to the interactions between AuNPs and WS_2, suggesting that AFW nanocomposite has been successfully prepared.

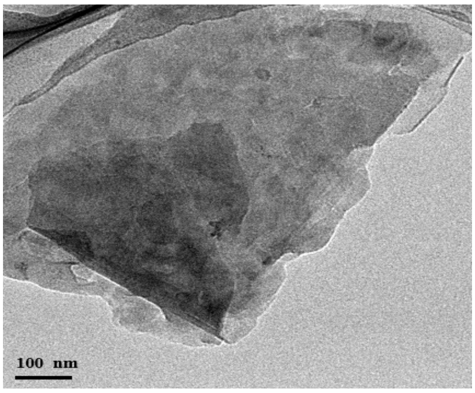

Figure S2. TEM images of bulk WS_2 after sonication

3.4 Quantitative analytical performance

Under optimal conditions, the amperometric response of the dual-component immune sensor to both CEA and CA72-4 components was studied by DPV. As shown in Figure 6A, with the increase in antigen concentration, more CEA and CA72-4 molecules specifically bind to Ab_1 in the sensor. This will lead to more immune complexes on the electrode surface formed by sandwich immunoassays with ATG and AFW labeled CEA and CA72-4 secondary antibodies, respectively, contributing to a continuous increase in the DPV current signal. The results indicate that this dual-component immune sensor exhibits good linear relationships to CEA and CA72-4, which are 0.01~120 ng/mL and 0.05~35 U/mL, respectively. The linear equations are $I = -0.006\ C_{CEA} - 0.845$ and $I = -0.0154 C_{CA72\text{-}4} - 0.1575$, with the linear correlation coefficients of 0.9913 and 0.9871, respectively (Figure 6B,C). The detection limits of the immune sensor for CEA and CA72-4 are 0.003 ng/mL and 0.016 U/mL, with S/N = 3. As shown in **Table S1**, the detection limits of the biosensor for CEA and CA72-4 are comparable to or better than those of some of the previously reported sensors [40–45].

Table S1. Comparison of LOD about different CEA/CA72-4 detection methods

Material	Method	Target	Linear range	LOD	Reference
Ag@ZIF-67	Electrochemiluminescence	CEA	0.05-500 ng/ml	0.00453ng/ml	[40]
AuNP-ox-MWCNT	Electrochemiluminescence	CEA	10-100 ng/ml	0.76 ng/ml	[41]
AuNPs/covalent organicframeworks/Ab_2/Ab_1/covalent organic frameworks	Voltammetric	CEA	0.11-80 ng/ml	0.034 ng/mL	[42]
AuNPs-TB-GO	Differential pulse voltammetry	CEA	0.01-120 ng/ml	0.003 ng/ml	This work

Nuclepore track-etched polycarbonate membranesn with Oligo RNA aptamers	Amperometric	CA72-4	4-14 U/ml	4 U/ml	[43]
Nanoporous Au/Ab_1/Ab_2/polyani-Line-Au	Electrochemical impedance spectroscopy	CA72-4	2-200 U/ml	0.1U/ml	[44]
MWCNT/GO	Differential pulse voltammetry	CA72-4	2-80 U/ml	0.4U/ml	[45]
AuNPs-FMC-WS_2	Differential pulse voltammetry	CA72-4	0.05-35 U/ml	0.016 U/ml	This work
